# Association between Proton Pump Inhibitor Use and the Risk of Female Cancers: A Nested Case-Control Study of 23 Million Individuals

**DOI:** 10.3390/cancers14246083

**Published:** 2022-12-10

**Authors:** Nhi Thi Hong Nguyen, Chih-Wei Huang, Ching-Huan Wang, Ming-Chin Lin, Jason C. Hsu, Min-Huei Hsu, Usman Iqbal, Phung-Anh Nguyen, Hsuan-Chia Yang

**Affiliations:** 1 Health Personnel Training Institute, University of Medicine and Pharmacy, Hue University, Hue 491-20, Vietnam; 2 School of Health Care Administration, College of Management, Taipei Medical University, Taipei 11031, Taiwan; 3 International Center for Health Information Technology (ICHIT), College of Medical Science and Technology, Taipei Medical University, Taipei 106339, Taiwan; 4 Graduate Institute of Biomedical Informatics, College of Medical Science and Technology, Taipei Medical University, Taipei 106339, Taiwan; 5 Biomedical Informatics & Data Science (BIDS) Section, School of Medicine, Johns Hopkins University, 2024 E Monument St, Suite 1-200, Baltimore, MD 21205, USA; 6 Department of Neurosurgery, Shuang Ho Hospital, Taipei Medical University, New Taipei City 235041, Taiwan; 7 Taipei Neuroscience Institute, Taipei Medical University, Taipei 110301, Taiwan; 8 Clinical Data Center, Office of Data Science, Taipei Medical University, Taipei 106339, Taiwan; 9 Clinical Big Data Research Center, Taipei Medical University Hospital, Taipei 110301, Taiwan; 10 Research Center of Health Care Industry Data Science, College of Management, Taipei Medical University, Taipei 110301, Taiwan; 11 International Ph.D. Program in Biotech and Healthcare Management, College of Management, Taipei Medical University, Taipei 110301, Taiwan; 12 Office of Data Science, Taipei Medical University, Taipei 110301, Taiwan; 13 Graduate Institute of Data Science, College of Management, Taipei Medical University, Taipei 110301, Taiwan; 14 Health ICT, Department of Health, Hobart, TAS 700, Australia; 15 Global Health and Health Security Department, College of Public Health, Taipei Medical University, Taipei 11031, Taiwan; 16 Research Center of Big Data and Meta-Analysis, Wan Fang Hospital, Taipei Medical University, Taipei 116079, Taiwan

**Keywords:** proton pump inhibitor, cancer risk, breast cancer, cervical cancer, endometrial cancer, ovarian cancer

## Abstract

**Simple Summary:**

The safety of long-term PPI use has increasingly raised concerns. We conducted a case-control study to explore the associations of PPI use with female cancer risks in specific age groups. Overall, PPI use was significantly associated with decreased risks of breast, cervical, endometrial, and ovarian cancers. PPIs were associated with a significant decrease in breast and ovarian cancer risks in 20–64-year-old users and a reduction in cervical and endometrial cancer risks in those aged 40–64 years. We hope that our findings based on real-world big data can provide researchers and clinicians with some possible insights. Further clinical studies are needed to elucidate the effects of PPIs on female cancers.

**Abstract:**

Background: Firm conclusions about whether long-term proton pump inhibitor (PPI) drug use impacts female cancer risk remain controversial. Objective: We aimed to investigate the associations between PPI use and female cancer risks. Methods: A nationwide population-based, nested case-control study was conducted within Taiwan’s Health and Welfare Data Science Center’s databases (2000–2016) and linked to pathologically confirmed cancer data from the Taiwan Cancer Registry (1979–2016). Individuals without any cancer diagnosis during the 17 years of the study served as controls. Case and control patients were matched 1:4 based on age, gender, and visit date. Conditional logistic regression with 95% confidence intervals (CIs) was applied to investigate the association between PPI exposure and female cancer risks by adjusting for potential confounders such as the Charlson comorbidity index and medication usage (metformin, aspirin, and statins). Results: A total of 233,173 female cancer cases were identified, consisting of 135,437 diagnosed with breast cancer, 64,382 with cervical cancer, 19,580 with endometrial cancer, and 13,774 with ovarian cancer. After matching each case with four controls, we included 932,692 control female patients. The number of controls for patients with breast cancer, cervical cancer, endometrial cancer, and ovarian cancer was 541,748, 257,528, 78,320, and 55,096, respectively. The use of PPIs was significantly associated with reduced risk of breast cancer and ovarian cancer in groups aged 20–39 years (adjusted odds ratio (aOR): 0.69, 95%CI: 0.56–0.84; *p* < 0.001 and aOR: 0.58, 95%CI: 0.34–0.99; *p* < 0.05, respectively) and 40–64 years (aOR: 0.89, 95%CI: 0.86–0.94; *p* < 0.0001 and aOR: 0.87, 95%CI: 0.75–0.99; *p* < 0.05, respectively). PPI exposure was associated with a significant decrease in cervical and endometrial cancer risks in the group aged 40–64 years (with aOR: 0.79, 95%CI: 0.73–0.86; *p* < 0.0001 and aOR: 0.72, 95%CI: 0.65–0.81; *p* < 0.0001, respectively). In contrast, in elderly women, PPI use was found to be insignificantly associated with female cancers among users. Conclusions: Our findings, based on real-world big data, can depict a comprehensive overview of PPI usage and female cancer risk. Further clinical studies are needed to elucidate the effects of PPIs on female cancers.

## 1. Introduction

Cancer is the leading cause of premature death and disability worldwide, particularly in women, with more than one out of every six deaths due to cancer [1]. Breast cancer has become the most prevalent of all female cancers, consisting of 12% of all new annual cancer cases globally. It is reported as the main cause of death among women [2,3]. Breast cancer is followed by cervical and ovarian cancers as common cancers, with 0.6 million and 313,959 new cases, respectively, diagnosed in 2020 [4,5]. Ovarian cancer was reported to be associated with the highest mortality of gynecological malignancies because of its silent development and advanced stage at diagnosis [6,7,8]. Endometrial cancer is the sixth most common cancer in women. In 2020, there were more than 417,000 new cases of endometrial cancer, and it was the 15th most common cancer overall [9]. 

The use of proton pump inhibitor (PPI) medications has rapidly increased in recent years because of their effectiveness in treating gastroesophageal reflux disease and peptic ulcer disease. Since their introduction in the late 1980s, millions of people have been using these drugs continuously or for long-term periods [10]. Studies have investigated the appropriateness and judiciousness of taking PPIs in the hospital and outpatient practices [11,12]. In addition, research on the association between female cancer risks such as breast, cervical, endometrial, and ovarian cancers and PPI use has been proposed. Studies have shown inconsistent results. Some suggested associations of PPIs with a decreased risk of breast cancer [13,14,15], whereas some concluded no significant association of their use with breast and endometrial cancers [16]. Some evidence has indicated that PPIs could suppress the growth of breast, cervical, endometrial, and ovarian cancer cells in vitro and in vivo [17,18,19]. Thus, the safety of long-term PPI use has increasingly raised concerns [20].

In Taiwan in 2019, cancers of the uterine body, ovaries and other adnexa, and cervix uteri ranked fifth, seventh, and ninth, respectively, among female cancers with the highest incidence rates, with a median age of incidence at 56, 54, and 57 years, respectively [21]. The incidence rates of uterine body and ovary cancers peaked at 50 and 60 years, while cervix uteri cancer climbed with age until 80 years [21]. However, to our knowledge, no studies have been conducted on the risks of female cancers among PPI users and included stratification by age. Therefore, this study aims to explore the associations of PPI use with female cancer risks in specific age groups.

## 2. Methods

### 2.1. Data Source

**Figure 1:**
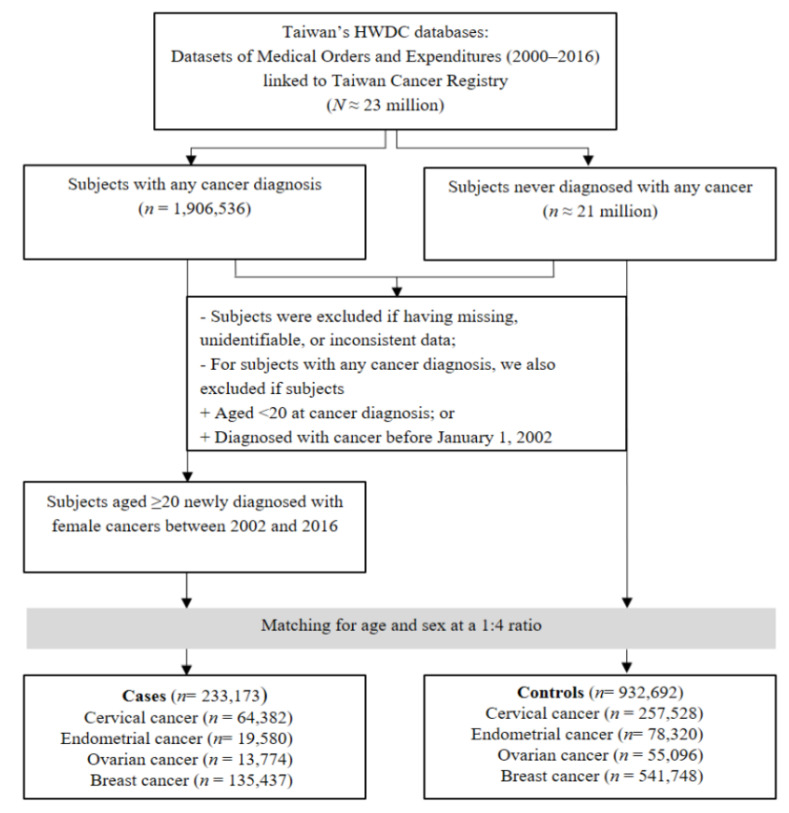
Workflow of the case-control study design.

We used Taiwan’s Health and Welfare Data Science Center (HWDC) databases, from which we retrieved medication and diagnosis data (2000–2016) and linked these to pathologically confirmed cancer data from the Taiwan Cancer Registry (TCR) (1979–2016) (Figure 1). The TCR is a population-based cancer registry standardizing medical definitions and terminology as well as codes and procedures of the registry’s reporting system that tracks patients with a cancer diagnosis. The HWDC is a centralized data repository administered by Taiwan’s Ministry of Health and Welfare (MOHW) that stores de-identified claims data of beneficiaries of the National Health Insurance (NHI) [22]. Taiwan’s NHI, as compulsory health insurance, covers 99.8% of the Taiwanese residents, accounting for over 23 million people [23]. The diagnoses of diseases in this study were identified from the validated International Classification of Diseases, Clinical Modification, Ninth Revision [ICD-9-CM] codes [22,23]. We obtained ethical approval for this study from the Joint Institutional Review Board of Taipei Medical University (TMU-JIRB), Taipei, Taiwan (approval number: N202003609).

### 2.2. Study Population

We conducted a case-control study using incident cases by extracting all newly diagnosed individuals with female cancers (e.g., ICD-9-CM codes 174 for breast, 180 for cervical, 182 for endometrial, and 183 for ovarian cancer) between 1 January 2002 and 31 December 2016. Those eligible patients were confirmed by the TCR database and defined as cases, and the date of a cancer diagnosis was defined as the index date. Individuals without any cancer diagnosis during the 17 years of the study served as controls. We randomly selected four controls among individuals for each case. Propensity score matching was applied for age, month, and year of diagnosis. Afterward, controls were assigned an index date corresponding to the diagnosis date of each case [24]. We excluded patients with cancer who were younger than 20 years of age and those who had missing, unidentifiable, or inconsistent data in this study.

### 2.3. PPI Exposure 

Information about patients’ medications was retrieved from the prescription claims in the HWDC database. We collected medication information including drug codes, drug names, dispensing data, and the total daily dose for each prescription. PPIs were classified using Anatomical Therapeutic Chemical (ATC) code A02BC (see Appendix A). PPI exposure was analyzed only before the cancer diagnosis (e.g., index date). We considered whether patients had ever been exposed to PPI medications or not. Thus, patients with PPI prescriptions prescribed for at least 60 days within two years before the index date were classified as PPI users. In addition, patients who had never been prescribed any PPIs were defined as non-users.

### 2.4. Confounding Factors

The propensity score was determined using logistic regression, as proposed by Rosenbaum and Rubin (1983) [25], to estimate the probabilities of patients between cancer (case) and non-cancer (control) groups, as shown in Table 1. A number of potential confounding factors were included in the study. The known or suspected use of drugs can modify the risk of cancers or influence carcinogenic effects, including metformin (ATC, A10BA02) [26,27,28], aspirin (ATC, B01AC06) [28,29,30], and statins (ATC, C10AA) [31] (Table 1). Exposure to those drugs was defined if they were prescribed for at least two months (e.g., 60 days) within two years before the index date.

The competing risks could confound the chance of cancer; thus, we identified those comorbidities that might be associated with mortality based on diagnostic codes from outpatient and hospitalization data. Charlson comorbidities were included in the analysis, except for cancer. Those diseases were considered if patients were administrated at least twice two years before the index date.

### 2.5. Statistical Analysis

Conditional logistic regression with 95% confidence intervals (CIs) was applied to investigate the association between PPI exposure and cancer risk. The models were adjusted for those potential confounding factors in Table 1 and stratified by different age groups (e.g., young age, 20–39; middle-aged, 40–64; elderly, more than 65 years; and overall age groups). All data management was performed using SAS v.9.4 software (SAS Institute Inc., Cary, NC, USA). Statistical analysis was 2-sided, and a *p*-value < 0.05 indicated statistical significance.

## 3. Results

### 3.1. Baseline Characteristics

We identified 1,906,536 patients newly diagnosed with cancer from 2000 to 2016. A total of 233,173 patients newly diagnosed with a female cancer at an age of 20 years or older between 2002 and 2016 were included as cases; these included 135,437 patients diagnosed with breast cancer, 64,382 with cervical cancer, 19,580 with endometrial cancer, and 13,774 with ovarian cancer (Figure 1). After matching each case with four controls for age and sex, we included a total of 932,692 control female patients, and the number of controls for patients with breast, cervical, endometrial, and ovarian cancers was 541,748, 257,528, 78,320, and 55,096, respectively. Both cancer cases and controls had an average age of 52.05 years (Table 1). The 40–64 age group predominated in all four cancers, accounting for 66.96%. The prevalence of peptic ulcer disease (25,949/233,173) and diabetes (120,765/932,692) was the highest in the case group and was lower than the figures for control group by 1.82% and 2.09%, respectively. The frequency of metformin, aspirin, and statin use in the case group was lower than that in the control group by 2.13%, 1.26%, and 1.86%, respectively.

### 3.2. Associations of PPI Use with Overall Female Cancers 

**Figure 2:**
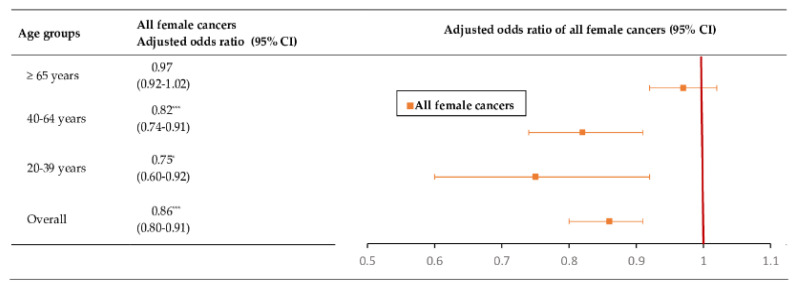
The association of PPI use with overall risk of all female cancers by age groups with adjusted odds ratios. CI, confidence interval. Footnote: * *p* < 0.05, *** *p* < 0.0001.

Figure 2 demonstrates the association between PPI use and female cancers among different age groups. Overall, PPI use was associated with a statistically significantly reduced risk of female cancers (adjusted odds ratio (aOR): 0.86, 95%CI: 0.80–0.91; *p* < 0.0001). The decrease in female cancer risk was found to be significantly associated with PPI users aged 20–39 years (aOR: 0.75, 95%CI: 0.60–0.92; *p* < 0.05) and 40–64 years (aOR: 0.82, 95%CI: 0.74–0.91; *p* < 0.0001); however, there was no significant association between PPI use and female cancer risk among those aged 65 years and older.

### 3.3. Associations of PPI Use with Breast, Cervical, Endometrial, and Ovarian Cancers 

**Figure 3:**
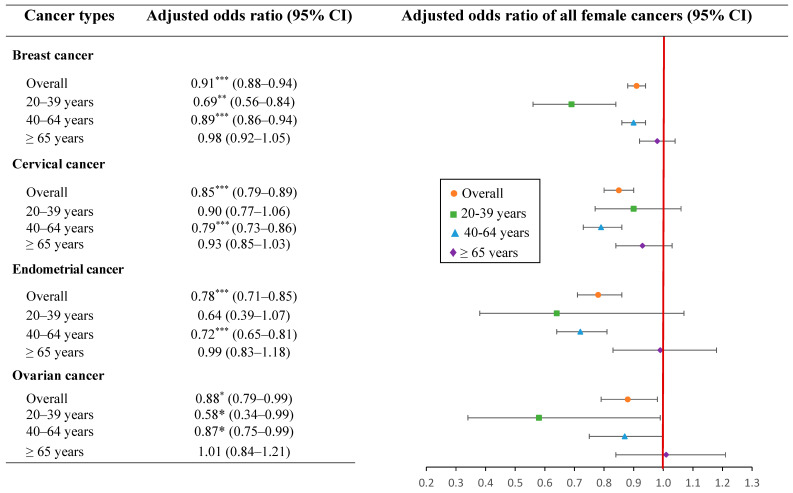
The association of PPI use with breast, cervical, endometrial, and ovarian cancer risks by age groups with adjusted odds ratios. CI, confidence interval. Footnote: * *p* < 0.05, ** *p* < 0.001, *** *p* < 0.0001.

Figure 3 presents the breast, cervical, endometrial, and ovarian cancer risks among PPI users by age group. The use of PPIs was significantly associated with a reduced risk of breast and ovarian cancers in the groups aged 20–39 years (aOR: 0.69, 95%CI: 0.56–0.84; *p* < 0.001 and aOR: 0.58, 95%CI: 0.34–0.99; *p* < 0.05, respectively) and 40–64 years (aOR: 0.89, 95%CI: 0.86–0.94; *p* < 0.0001 and aOR: 0.87, 95%CI: 0.75–0.99; *p* < 0.05, respectively). PPI exposure was associated with a significant decrease in cervical and endometrial cancer risks in the group aged 40–64 years (aOR: 0.79, 95%CI: 0.73–0.86; *p* < 0.0001 and aOR: 0.72, 95%CI: 0.65–0.81; *p* < 0.0001, respectively). In addition, PPI use was associated with a lower risk of breast, cervical, endometrial, and ovarian cancers among females aged ≥65 years, but no significant association was found between these cancer risks and PPI use. Likewise, there was no statistically significant association between PPI use in the 20–39 age group and cervical and endometrial cancer risks.

## 4. Discussion

### 4.1. Main Findings

In this case-control study, we demonstrated an association between PPI use and risks of female cancers. PPI use was associated with a significant decrease in breast and ovarian cancers in users aged 20–64 years and a reduction in cervical and endometrial cancer risks in those aged 40–64 years. In contrast, in elderly women, our findings found that there was an insignificant association between PPI users and female cancer risks.

### 4.2. Biological Plausibility

#### 4.2.1. Breast Cancer

As found in this study, the use of PPIs was linked to a lower risk of breast cancer. Probable mechanisms have been proposed to elucidate the potential antitumor activity of PPIs. First, the inhibition of the H+/K+-ATPase might contribute to the build-up of protons inside cells to reduce the intracellular pH, which prevents the development of breast cancer cells [32,33]. The association between esomeprazole, a PPI, and melanoma cell inhibition through a caspase-dependent pathway involving cytosolic acidification and alkalinization of the tumor pH was indicated in an in vitro study [34]. In addition, PPIs can directly suppress cancer development by targeting tumor-specific T cell-originated protein kinase through a proton pump-independent mechanism [35]. Second, PPIs also act as vacuolar H+-ATPase (V-ATPase) inhibitors, suggesting that they might affect the tumor acidic microenvironment and prevent the extracellular signal that controls the activity of kinase 1/2, Akt/Src kinases, and pyruvate kinase M2 from being phosphorylated. As a result, cancer cell growth might be inhibited or undergo apoptosis [36,37,38]. Ihraiz et al. (2020) investigated the effects of PPIs in three breast cancer cell lines including MCF-7, T47D, and MDA-MB-231 and revealed that PPI treatment was significantly associated with a decrease in breast cancer cells [15].

#### 4.2.2. Cervical Cancer

Little evidence has indicated the direct mechanism of the association between PPI use and cervical cancer; however, regarding the treatment mechanism, PPI use increases the effectiveness of treatment, especially in resistant forms [19]. One of the major mechanisms might be that PPIs directly inhibit the V-ATPase, which plays a vital role in pumping protons across the plasma membrane and across the membranes of numerous intracellular compartments at the cellular level [19,39]. An in vitro study demonstrated the inhibition of the V-ATPase via siRNA or that PPIs might enhance the chemosensitivity of paclitaxel in cervical cancer cells [19]. Another mechanism is via fast intracellular acidification and activation of the caspase enzymes [37]. Lee et al. (2011) showed that omeprazole, a PPI, has the ability to mitigate the resistance of tumor cells to chemotherapy, by altering the process of the transfer of lysosomes, and activate programmed cell death mechanisms. In addition, other studies have indicated that a mixture of esomeprazole and amygdalin can suppress the development of cervical cancer cells in vitro [40,41].

#### 4.2.3. Endometrial Cancer

The results highlighted that PPI use was statistically significantly associated with a decreased risk of endometrial cancer. There are two probable mechanisms that may explain our finding. First, PPIs induce chemosensitization and/or have an impact because of direct pH-dependent antitumor activity that might mediate the beneficial effects of PPIs [33]. Previous studies have indicated the possible antitumor impacts of PPIs [42,43,44]. Second, PPI treatment inhibits proliferation of cancer by inhibiting V-ATPases residing in the plasma membrane, including intracellular acidification and alkalization of the tumor microenvironment, which have a chemopreventive impact [45]. Numerous in vitro and in vivo studies have investigated the impacts of V-ATPase activity on several cancers such as pancreatic, breast, cervical, and prostate cancers and malignant melanomas [19,41,45,46,47]. It is therefore conceivable that PPIs block the V-ATPase to ultimately enhance cytotoxicity and apoptosis.

#### 4.2.4. Ovarian Cancer

Regarding ovarian cancer risk in PPI users, our findings indicated a decreased risk of ovarian cancer in PPI users. Some possible mechanisms support our finding. First, the acidic microenvironment of cancer cells has been shown to be correlated with cancer aggressiveness, including increased invasiveness, angiogenesis, metastasis, and chemoresistance [48]. Furthermore, extracellular acidity suppresses the activity of cytotoxic T lymphocytes and natural killer cells, consequently decreasing antitumor defenses [49]. Tumors have adapted to acidic microenvironments through overexpression of proton pumps, which extrude protons out from the intracellular space of tumor cells. Earlier studies have demonstrated this phenomenon in numerous cell lines, including ovarian adenocarcinomas [19,50]. Second, the inhibition of V-ATPases reduces the acidity of the tumor microenvironment, which slows cell proliferation and triggers tumor cell apoptosis. Hence, PPIs might have antitumor activity and enhance the effectiveness of antitumor therapy through V-ATPase inhibition [51,52]. An in vitro study highlighted that omeprazole, a PPI, enhanced the impact of chemotherapeutic agents on chemoresistant epithelial ovarian cancer and clear cell carcinoma by reducing the acidic tumor microenvironment [17]. Furthermore, Lee et al.’s study (2015) revealed that elevated expression of V-ATpase mRNA was found to be significantly associated with poor survival in patients with ovarian cancer. Third, PPIs could potentially inhibit fatty acid synthase (FASN) using the crystal structure of FASN thioesterase, inducing apoptosis in chemosensitive and platinum-resistant ovarian cancer cells. PPI inhibition of FASN has been shown in in vitro and in vivo studies [53,54,55].

Interestingly, our results revealed that a significantly reduced risk of breast cancer was observed in PPI users aged 20–64 years, which was consistent with the findings of previous case-control studies [13,56,57]. In addition, evidence from an age stratification analysis in a cohort study indicated that the benefit increased with age, especially among older PPI users aged 50–65 years [58]. An Icelandic population-based case-control study, nevertheless, found no significant association between PPI use and breast cancer. This inconsistency could be due to the study population, sample size, and adjusted confounders [45]. Likewise, the decrease in ovarian cancer risk was significant in 20–64-year-old PPI users. Indeed, a previous study indicated that PPIs directly bind to the active site and inhibit FASN thioesterase, providing a crucial foundation for repositioning PPIs as anticancer treatments [53]. Regarding long-term and high-dose PPI treatment that has been demonstrated to be well tolerated in patients with few side effects, repositioning PPIs as anticancer medications will unlikely be associated with increased toxicity [10,59]. In terms of cervical and endometrial cancers, our findings showed that PPI drug use was associated with a significantly decreased cancer risk in females aged 40–64 years. Ballinger et al. (2022) conducted an observational study and clinical trials on postmenopausal women and demonstrated that taking PPI medications was not related to endometrial cancer; however, they found a trend in decreased risk with increasing PPI potency. Inconsistencies between our study and previous observational studies might be attributed to the study period, number of subjects, and adjusted confounders [16].

## 5. Strengths and Limitations

Our study possesses a number of strengths. This study features its high-quality registry data. Not only were all PPIs in Taiwan recorded in the HWDC databases, but cancer cases were also identified based on the Taiwan Cancer Registry database where all cancer diagnoses have been confirmed by pathology. Furthermore, the extensive database consisting of 23 million patients’ claims data enabled us to stratify subjects by age. To our knowledge, this current study is the first to involve a subgroup age analysis for female cancers and PPIs. Although a previous relevant study stratified individuals by age, it analyzed the association of PPI use with breast cancer risk rather than the risk of cervical, endometrial, and ovarian cancer risk [58].

We acknowledge that our study has its limitations. First, this study revealed associations instead of causality between PPIs and cancer risks. This study preliminarily showed potential cancer medication signals for clinicians or researchers to conduct and determine their causality or mechanisms in the future” to this sentence “This study preliminarily showed potential PPI medication signals and female cancer risks for clinicians or researchers to conduct and determine their causality or mechanisms in the future. Second, patient’ lifestyles, medication adherence, PPI dosage, and laboratory data were not provided in the HWDC database. Despite the unavailability of such data, all cancer diagnoses in our study were confirmed based on pathological reports, and the large sample size of millions of individuals could mitigate the impact caused by the lack of adherence data. Third, some of the established risk factors were not extracted from the HWDC database, such as hormone replacement therapy, oral contraception, obesity (e.g., BMI), HPV infection or vaccination, hypertension, hyperinsulinemia, and number of pregnancies/infertility, etc. Fourth, the other limitation is the retrospective format of the study. Finally, the results of this study cannot be generalized to other populations.

## 6. Conclusions

Overall, PPI use was significantly associated with decreased risks of breast, cervical, endometrial, and ovarian cancers. PPIs were associated with a significant decrease in breast and ovarian cancer risks in 20–64-year-old users and a reduction in cervical and endometrial cancer risks in those aged 40–64 years. Notably, our results should be interpreted with concern, because they demonstrate associations but not causality between PPI use and female cancer risks. We hope that our findings based on real-world big data can provide researchers and clinicians with some possible insights. Further clinical studies are needed to elucidate the effects of PPIs on female cancers.

## Figures and Tables

**Table 1:** Baseline characteristics of cases and controls for female cancers.

Characteristics	Cases (with Cancer) (n = 233,173)	Controls (without Cancer) (n = 932,692)	*p*-Value
**Age**			
Mean ± SD	52.05 ± 12.75	52.05 ± 12.74	1
20–39 years, n (%)	36,968 (15.85)	147,872 (15.85)	1
40–64 years, n (%)	156,120 (66.96)	624,480 (66.96)	1
≥65 years, n (%)	40,085 (17.19)	160,340 (17.19)	1
**Comorbid conditions, *n* (%)**			
Myocardial infarction	421 (0.18)	1921 (0.21)	0.015
Congestive heart failure	3202 (1.37)	14,451 (1.55)	<0.0001
Peripheral vascular disease	1551 (0.67)	7319 (0.78)	<0.0001
Cerebrovascular disease	8985 (3.85)	41,794 (4.48)	<0.0001
Dementia	1583 (0.68)	7660 (0.82)	<0.0001
Chronic pulmonary disease	6538 (2.8)	29,240 (3.14)	<0.0001
Rheumatic disease	3140 (1.35)	15,928 (1.71)	<0.0001
Peptic ulcer disease	25,949 (11.13)	120,765 (12.95)	<0.0001
Liver disease	12,912 (5.54)	59,355 (6.36)	<0.0001
Diabetes	29,356 (12.59)	141,444 (15.17)	<0.0001
Hemiplegia or paraplegia	352 (0.15)	1742 (0.19)	<0.001
Renal disease	4876 (2.09)	22,314 (2.39)	<0.0001
**CCI**			
Mean ± SD	0.47 ± 0.92	0.53 ±0.94	
**Other drugs, *n* (%)**			
Metformin	21,758 (9.33)	106,760 (11.46)	<0.0001
Aspirin	15,508 (6.65)	73,217 (7.91)	<0.0001
Statin	17,244 (7.40)	86,129 (9.26)	<0.0001

CCI, Charlson comorbidity index.

## Data Availability

Restrictions apply to the availability of these data. Data were obtained from databases of Health and Welfare Data Science Center and are available with the permission of Taiwan’s Ministry of Health and Welfare.

## Abbreviations

aOR	Adjusted odds ratio
ATC Classification	Anatomical Therapeutic Chemical classification
CCI	Charlson comorbidity index
CI	Confidence interval
FASN	Fatty acid synthase
HWDC	Health and welfare data science
ICD-9-CM	International Classification of Diseases, 9th revision, Clinical Modification
MOHW	Ministry of Health and Welfare
NHI	National health insurance
PPI	Proton pump inhibitor
TMU-JIRB	Taipei Medical University—Joint Institutional Review Board
TCR	Taiwan Cancer Registry
V-ATPase	Vacuolar H+-ATPase

## Appendix A

**Table A1:** The PPIs classification using Anatomical Therapeutic Chemical (ATC) code A02BC.

ATC Code	Name	Available in Taiwan
A02BC01	omeprazole	1995~
A02BC02	pantoprazole	1998~
A02BC03	lansoprazole	2004~
A02BC04	rabeprazole	2000~
A02BC05	esomeprazole	2002~
A02BC06	dexlansoprazole	2004~
A02BC07	dexrabeprazole	Not Available
A02BC08	vonoprazan	Not Available
A02BC09	tegoprazan	Not Available
